# HIF1 inhibition targets tumoral and myeloid cells, and is a promising therapy for metastatic castration-resistant prostate cancer

**DOI:** 10.1038/s41419-026-08590-8

**Published:** 2026-03-27

**Authors:** Darya Yanushko, Angélique Pichot, Alexandre Vincent, Céline Keime, Gilles Laverny, Daniel Metzger

**Affiliations:** 1 Institut de Génétique et de Biologie Moléculaire et Cellulaire, Illkirch, France; 2 Centre National de la Recherche Scientifique (CNRS), UMR7104, Illkirch, France; 3 Institut National de la Santé et de la Recherche Médicale (INSERM), U1258, Illkirch, France; 4 Université de Strasbourg, Strasbourg, France

**Keywords:** Cancer microenvironment, Cancer models, Metastasis

## Abstract

Metastatic prostate cancer (PCa) remains lethal due to limited effective therapies. *PTEN* and *TP53* are commonly mutated tumor suppressors in metastatic PCa, yet the molecular and cellular mechanisms driving aggressiveness and treatment resistance are not fully understood. We have previously shown that mice with both *Pten* and *Trp53* inactivation in prostate luminal cells at adulthood develop aggressive intraductal prostate carcinoma (IDC) and liver metastases. By combining single-cell and spatial transcriptomics, along with flow cytometry and immunohistochemical analyses, we now reveal that prostatic tumors of such mice are hypoxic and progress within a complex immune microenvironment, including neutrophils, TREM2⁺ macrophages, and CCR2⁺ myeloid cells. Moreover, we uncovered that genetic *Hif1a* inactivation in prostate luminal cells or pharmacological inhibition of HIF1 signaling in Pten/Trp53^(i)pe−/−^ mice does not prevent IDC formation or epithelial plasticity driven by *Trp53* loss, but impairs neutrophil recruitment. In addition, HIF1 inhibition reduces CCR2⁺ myeloid cell infiltration. Importantly, targeting these immune cells sensitizes tumors to androgen deprivation and reduces the size of liver metastatic niches. Moreover, pharmacological HIF1 inhibition not only overcomes castration resistance, but also eliminates metastatic niches, offering a more effective approach than direct myeloid cell blockade. Thus, HIF1 targeting emerges as a promising therapy for metastatic castration-resistant PCa.

## Introduction

Prostate cancer (PCa) is the most frequent male malignancy in western societies and the second leading cause of cancer-related deaths [1]. Localized disease is of favorable prognosis, in contrast to metastatic forms [2]. Indeed, while initially responding to androgen-deprivation therapy, most PCa progress to an androgen-independent state and develop resistances to currently available therapeutic options [3]. Tumors are infiltrated by a large number of cells with immunosuppressive characteristics, but few T-cells, and clinical trials have shown poor responsiveness to immune checkpoint treatments [4, 5]. De novo metastatic PCa is characterized by a more aggressive course with a shorter time of onset of castration resistance and worse overall survival than metachronous metastatic disease [6]. It accounts for about 5% of all prostate cancer diagnoses, but is responsible for nearly 50% of PCa-related deaths [7]. Thus, unraveling factors contributing to the initiation of the metastatic cascade, dissemination to distant organs and therapy resistance is of utmost importance to design novel therapeutic strategies and improve patients’ survival.

Metastatic PCa is associated with an enrichment of driver mutations of the *PTEN* and *TP53* tumor suppressor genes [8]. Multiple studies characterized cell-autonomous effects of such mutations on tumor biology, including increased proliferation, DNA damage response, inflammation and epigenetic plasticity [9]. Recent efforts in single-cell analyses provided an extensive characterization of cell types present in the tumor microenvironment, and their contribution to tumor progression through secretion of signaling molecules, extracellular matrix remodeling and immunosuppression. Moreover, recent studies showed that immunosuppressive myeloid cells promote prostate cancer progression [10–13]. In addition, hypoxia has recently been shown to be associated with a particular histological subtype of prostate cancer, namely intraductal carcinoma (IDC) [14, 15], an aggressive tumor subtype (Gleason grade 4) with an increased risk of metastasis [16, 17].

We previously uncovered hypoxia as an early feature of PTEN-loss induced prostatic intraepithelial neoplasia (PIN) formation, and hypoxic signaling as a driver of an immunosuppressive microenvironment and IDC formation, as well as castration resistance [18, 19]. Importantly, IDC of Pten/Trp53^(i)pe−/−^ mice in which both *Pten* and *Trp53* are selectively inactivated in prostatic epithelial cells (PECs) develop more rapidly than in Pten^(i)pe−/−^ mice [20], and disseminate in the liver [21]. They are characterized by increased epithelial cell plasticity induced by a crosstalk with cancer-associated fibroblasts (CAFs), which in turn enhances STAT3 signaling in PECs, leading to a partial Epithelial-to-Mesenchymal Transition (EMT) [21].

However, whether hypoxia also influences the tumor microenvironment in PTEN/p53-deficient tumors and contributes to metastatic progression of IDC is currently unknown. In this study, we investigate the molecular and cellular mechanisms underlying PCa progression, castration resistance and metastatic dissemination in Pten/Trp53^(i)pe−/−^ mice.

## Materials and methods

### Study design

The objective of the study was to characterize the role of hypoxia in metastatic PCa progression. To this aim, we generated Pten/Trp53^(i)pe−/−^, Pten/Trp53/Hif1a^(i)pe−/−^, and control mice that were randomized and subjected to various treatments. Only male mice were analyzed, as prostate cancer modeling is relevant only in animals of this sex. Investigators were not blinded to animals’ genotypes or treatment regimens. The impact of various treatments on prostate tumors was determined by one investigator. Sample size for animal experiments was estimated using EDA (NC3R) assuming 10% of interindividual variability and 30% of observed effect. Pten/Trp53^(i)pe−/−^ mice that developed sarcomatoid tumors [21] were excluded from the study.

### Mice

PSA-CreER^T2(tg/0)^/Pten^L2/L2^/Trp53^L2/L2^ mice were intercrossed with Hif1a^L2/L2^ mice in which *Hif1a* alleles are floxed [22], to generate PSA-CreER^T2(tg/0)^/Pten^L2/L2^/Trp53^L2/L2^/Hif1a^L2/L2^ and PSA-CreER^T2(0/0)^/Pten^L2/L2^/Trp53^L2/L2^/Hif1a^L2/L2^ mice. Pten/Trp53^(i)pe−/−^ and Pten/Trp53/Hif1a^(i)pe−/−^ male mice, as well as sex-matched Pten/Trp53^L2/L2^ and Pten/Trp53/Hif1a^L2/L2^ control littermates (all on a C57BL/6 genetic background) were generated by intraperitoneal injection of Tamoxifen (1 mg/mouse/day) for 5 days to 8 to 10 week old PSA-CreER^T2(Tg/0)^/Pten^L2/L2^/Trp53^L2/L2^, PSA-CreER^T2(Tg/0)^/Pten^L2/L2^/Trp53^L2/L2^/Hif1a^L2/L2^, PSA-CreER^T2(0/0)^/Pten^L2/L2^/Trp53^L2/L2^ and PSA-CreER^T2(0/0)^/Pten^L2/L2^/Trp53^L2/L2^/Hif1a^L2/L2^ mice, respectively, as described [23].

### Mouse treatments

For hypoxia determination, mice were intraperitoneally injected with pimonidazole HCl (60 mg/kg) (HP1-100Kit) following the manufacturer’s instructions and euthanized 90 min later. Prostates were collected and fixed following standard procedures.

PX-478 (TargetMol, T6961) was dissolved in water at a concentration of 8 mg/mL and administered to mice via oral gavage at a daily dose of 20 mg/kg. CCR2 antagonist (Santa Cruz Biotechnologies, sc-202525) was dissolved in 10% DMSO, 10% Tween 80 and 80% saline (0.9% NaCl) at a concentration of 20 µg/mL and administered by intraperitoneal injection at 50 µg/kg every 2 days for 2 weeks. SX682 (MedChemExpress, HY-119339) was dissolved in sunflower oil at a concentration of 20 mg/mL and administered at 50 mg/kg by oral gavage twice a day for 1 week.

Bilateral orchiectomy was performed under aseptic conditions by trained operators. Control animals underwent an abdominal laparotomy without resection of testicles (sham operation). Surgery was performed under anesthesia with isoflurane (4%). Mice were intraperitoneally treated with buprenorphine (0.1 mg/kg) before surgery and with metacam (1 mg/kg) after surgery to prevent pain.

### Histological examination

Five µm histological sections were prepared from paraffin embedded tissues and dried overnight at 37 °C. Hematoxylin and eosin (H&E) staining was performed according to standard protocols. Slides were scanned in brightfield mode with an AxioScan 7 digital slide scanner (Zeiss). Gland area and cell density within prostate glands were analyzed with the QuPath software [24]. Representative images are provided.

### Immunostaining

Five µm paraffin tissue sections were deparaffinized according to standard protocols and incubated for 20 min in Tris-EDTA Unmasking solution (Tris 10 mM, EDTA 1 mM, pH 9) for CCR2 and TREM2 immunostainings and in Citrate Unmasking Solution (Sodium Citrate 10 mM, Tween 20 0.05%, pH 6) for others, in a pressure cooker for heat-induced antigen retrieval. The following primary antibodies were used: Hypoxyprobe (Hypoxyprobe, HP1-100Kit, 1:50), HIF1A (Abcam, ab51608, 1:100), CXCL5 (Thermo Fisher Scientific, BS-2549R, 1:200), pSTAT3 (Tyr705) (CST, 9145S; 1:200), CCR2 (Abcam, ab273050, 1:200), pan-keratin (Proteintech, 26411-1-AP, 1:1500), F4/80 (CST, 70076S, 1:200), CD3 (Abcam, ab16669, 1:200), Ly6G (BioLegend, 127602, 1:50), TREM2 (R&D Systems, MAB17291, 1:100), and pAKT (S473) (CST, 4060, 1:200). For immunohistochemistry, one drop of SignalStain® Boost IHC Detection Reagent (CST 8114) was added to each section, and SignalStain® DAB Substrate Kit (CST, 8059) used to develop the signal, according to the manufacturer’s instructions. Sections were counterstained with hematoxylin and mounted. Images were acquired using an AxioScan 7 digital slide scanner (Zeiss). The percentage of positive cells within prostate glands was determined using the QuPath software [24].

For immunofluorescence, secondary antibodies coupled to Alexa Fluor 555 (Invitrogen, A31570, 1:400), Alexa Fluor 647 (Invitrogen, A32849, 1:400) or Alexa Fluor 488 (Invitrogen, A21206, 1:400) were used. Sections were mounted with Fluoromount-G^TM^ (Invitrogen, 00-4959-52). Images were acquired with a Leica DM 4000 B microscope equipped with a Photometrics CoolSNAP HQ2 camera.

### Flow cytometry

Prostates were collected, the dorso-lateral prostate (DLP) isolated, weighted, minced and incubated for 30 min at 37 °C with Liberase^TM^ TL 0.2 mg/mL (Roche, 05401020001) in 2 mL RPMI, 1% Penicillin/Streptomycin (P/S). Mechanical dissociation was performed with a gentleMACS™ Tissue Dissociator (Miltenyi). The cellular suspension was washed with 10 mL RPMI, 1% P/S, filtered through a 40 μm cell strainer and centrifuged. The pellet was resuspended in PBS 2% fetal calf serum (FCS). Cells were preincubated with anti-CD16/32 antibody (BD, 553141, 1:50) for 15 min and stained with antibodies in PBS 2% FCS at 4 °C for 30 min in the dark. Blood (30 µL) was collected in heparin/lithium-coated tubes (Microvette® 50. LH, Sarstedt, 20.1345.100) and mixed with 30 µL of antibodies (2x concentrated) for 30 min at RT in the dark. Red blood cells were lysed with 500 µL of FACS^TM^ Lysing Solution (BD, 349202) according to the manufacturer’s protocol.

The following antibodies were used: CD45 (Invitrogen, 56-0451-82, 1:100), CD11b (BioLegend, 101228, 1:100), Ly6C (BD Horizon^TM^, 562728, 1:100), Ly6G (BioLegend, 127645, 1:100), F4/80 (Invitrogen, 2611773, 1:100), CD206 (BioLegend, 141704, 1:100), Ter-119 (BioLegend, 116206, 1:250), CD31 (BioLegend, 102406, 1:250), CD326 (BioLegend, 118216, 1:100), CD49f (Invitrogen, 12-0495-83, 1:25), and Sca-1 (BD Horizon^TM^, 563990, 1:75). Viability was assessed with 4’,6-diamidino-2-phenylindole (DAPI) (BD Pharmingen™, 564907). Flow cytometry was performed on a FACS Fortessa X20 system (BD Biosciences) and data analyzed with the Flowjo software (Flowjo LLC). Doublets and dead cells were excluded from the analysis.

### Spatial transcriptomic analysis

Spatial transcriptomic was carried out using the Visium CytAssist Spatial Gene Expression for Formalin-Fixed and Paraffin-Embedded (FFPE) assay (10X Genomics, Pleasanton, CA, USA). FFPE sample passed the RNA quality control with a DV200 > 80%. Tissue section was cut at 5 µm from a FFPE tissue block using a Leica RM2165 microtome (Leica) and placed on a Superfrost Plus Epredia™ slide (Thermo Fisher Scientific). The tissue slide was dried for 3 h at 42 °C followed by overnight storage in a slide mailer containing a desiccant at room temperature (Demonstrated protocol CG000518 Rev. B, 10X Genomics). Subsequently, the section was deparaffinized and stained for H&E according to 10X Genomics recommendation (Demonstrated protocol CG000520 Rev. A) before imaging at 20X magnification using a Hamamatsu NanoZoomer 2.0HT digital slide scanner in raw data mode, generating a TIFF file. Stained sections were decrosslinked and processed with the mouse whole transcriptome probe panel (Mouse Visium transcriptome probes v1), as outlined in the demonstrated protocol « Visium CytAssist Spatial Gene Expression Reagent kits for FFPE, UserGuide » (Part number CG000495, Rev. C, 10X Genomics). The captured probes were used to prepare a gene expression library for sequencing. Quantification and quality control of the library were performed using a Bioanalyzer 2100 (Agilent Technologies, Santa Clara, CA). The library was sequenced on an Illumina NextSeq 2000 sequencer as paired-end 28 + 50 bp reads. RTA version 2.7.7 and BCL Convert version 3.8.4 were used to generate FASTQ files. Image and read processing were performed using Spaceranger v2.0.1 count with Visium_Mouse_Transcriptome_Probe_Set_v1.0_mm10-2020-A.csv probe set and refdata-gex-mm10-2020-A reference.

### scRNA-seq analysis

Mouse prostates were collected, and 25 mg of minced DLP were used for the single-cell analysis according to Demonstrated protocol CG000553 Rev. B (10X Genomics). After thawing, cell counting was performed using an ADAM MC Plus Fluorescence cell counter (NanoEntek, Seoul, Korea) with an Acridine Orange solution and DAPI. For each sample, between 506,000 and 1,540,000 cells were hybridized with a unique Mouse WTA Probe Barcode (10x Genomics, PN-1000492). After cell counting, 5000 cells per hybridization reaction were loaded onto the Chromium X using a Next GEM Chip Q. Single-cell libraries were generated using the Chromium Next GEM Single Cell Fixed RNA reagent kit (10x Genomics, Leiden, The Netherlands), according to the manufacturer’s recommendations. cDNA amplification was completed using 8 cycles of PCR. Dual-index library construction was performed with 9 cycles for the one library (SWWJ41/42/43/44) and 10 cycles for the other one (SWWJ31/32) during sample index PCR. Final libraries quantification and quality control were performed using the Bioanalyzer 2100 (Agilent Technologies, Les Ulis, France). Libraries were sequenced on an Illumina NextSeq 2000 sequencer as paired-end 28 + 85 bp reads. Image analysis and base calling were performed using RTA version 2.7.7 and BCL Convert version 3.8.4. Cell Ranger 7.2.0 count was used for alignment, barcode and UMI (Unique Molecular Identifier) filtering and counting, with Chromium_Mouse_Transcriptome_Probe_Set_v1.0.1_mm10-2020-A.csv probe set and refdata-gex-mm10-2020-A reference.

### Data analysis

Statistical analysis was performed using the GraphPad Software Prism 9.

Sequencing data are deposited at the Gene Expression Omnibus (GEO) database (GSE303530). The single-cell and spatial transcriptomic analyses are available at Github repository on demand.

## Results

### Hypoxic niches in PTEN- and p53-deficient prostatic tumors promote neutrophil infiltration

**Fig. 1:**
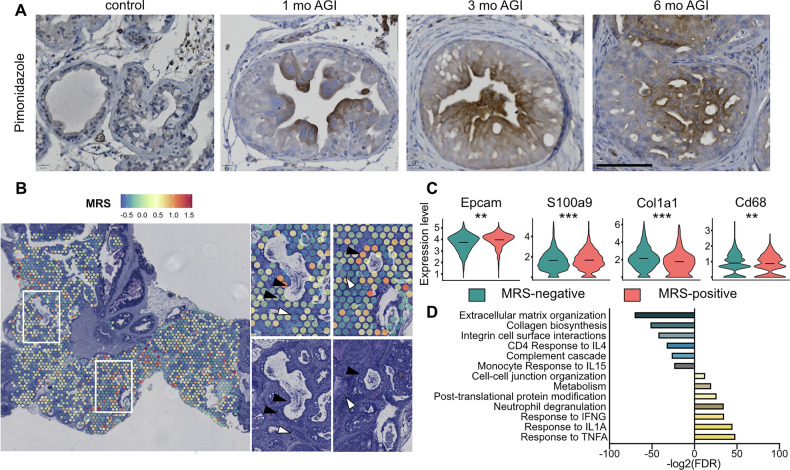
Characterization of hypoxic regions in prostatic tumors of Pten/Trp53^(i)pe−/−^ mice. **A** Representative immunohistochemical detection of pimonidazole in DLP of control (Pten^L2/L2^/Trp53^L2/L2^) and Pten/Trp53^(i)pe−/−^ mice 1, 3 and 6 months (mo) AGI. *n* ≥ 3 animals per group. Scale bar = 100 µm. **B** Spatial transcriptomic profile of the expression of modified Ragnum score (MRS) of a prostate section of a Pten/Trp53^(i)pe−/−^ mouse 6 months AGI. Higher magnifications of boxes area are shown on the right, as well as corresponding hematoxylin-eosin staining. Black arrows point to IDC, white arrows to stroma. **C** Violin plots of the transcript levels of the indicated genes determined by spatial transcriptomic analysis, segregated by the MRS. **, *p* < 0.01; ***, *p* < 0.001; Wilcoxon rank-sum test. **D** Pathway over-representation analysis (ORA) in MRS-negative regions (blue) and MRS-positive regions (yellow). FDR false discovery rate.

To determine the presence of hypoxic cells in PTEN- and p53-deficient prostatic tumors, we administered Pten/Trp53^(i)pe−/−^ mice with the hypoxia probe pimonidazole. PECs located in the vicinity of the lumen of PIN were immunostained 1 month after gene inactivation (AGI). Moreover, 3 and 6 months AGI most PECs were hypoxic in IDC (Fig. 1A).

To characterize hypoxic areas in IDC, we performed Visium spatial transcriptomic profiling of a prostate section of a Pten/Trp53^(i)pe−/−^ mouse 6 months AGI. We identified them by a hypoxia gene signature established in prostate cancer patients [25], from which genes associated with proliferation were excluded, as most PECs of Pten/Trp53^(i)pe−/−^ mice are senescent at this stage [21]. The resulting Modified Ragnum Signature (MRS; *Tdg, Ung, Xrcc6, Adm, Ddit4, Dsp, Fer1l4, Hilpda, P4ha1, Pkm, Rnase4, Rimkla, Spag4*) was detected in various prostatic area, and was enriched in tumor-containing regions [Fig. 1B (black arrows - tumor, white arrows–stroma), Supplementary Fig. 1A]. The analysis of differentially expressed genes (DEG) revealed that the transcript levels of the epithelial marker *Epcam* and of the neutrophil markers *S100a8* and *S100a9* were higher in regions with a positive MRS score (Fig. 1C; Supplementary Table 1). In addition, Gene Set Enrichment Analysis indicated that genes upregulated in regions with positive MRS score are associated with response to TNFa, IL1a and IFN [26] and neutrophil degranulation pathways (Fig. 1D, Supplementary Table 2), in agreement with neutrophil infiltration in IDC [21]. In contrast, Extracellular matrix organization, Collagen biosynthesis, Monocyte response to IL15 and Lymphoid cell activity pathways were enriched in regions with a negative MRS score (Fig. 1D, Supplementary Table 3), indicating that those regions correspond to stromal areas. In agreement, the expression of the fibroblast marker *Col1a1* and the macrophage marker *Cd68* was higher in those regions (Fig. 1C). Together, these results indicate that areas enriched in tumoral cells are characterized by transcriptional programs associated with hypoxia, and are within a complex microenvironment.

**Fig. 2:**
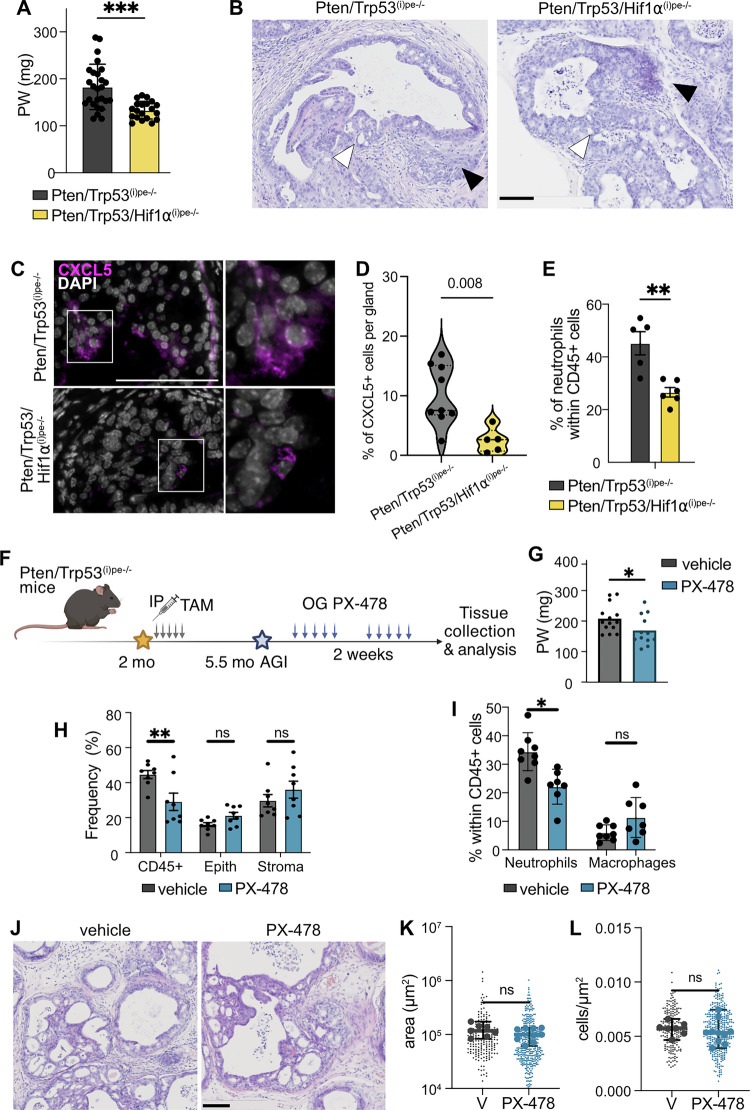
Characterization of the impact of genetic and pharmacological inhibition of HIF1a on tumors of Pten/Trp53^(i)pe−/−^ mice. **A** Prostate weight (PW) of Pten/Trp53^(i)pe−/−^ and Pten/Trp53/Hif1a^(i)pe−/−^ mice 5 months AGI. *** *p* < 0.001, unpaired Student t-test. **B** Representative H&E staining of DLP sections of Pten/Trp53^(i)pe−/−^ and Pten/Trp53/Hif1a^(i)pe−/−^ mice 5 months AGI. *n* ≥ 4 mice per group. Black arrows point to invasive regions, white arrows to cribriform regions. Scale bar = 100 µm. **C** Representative immunofluorescent staining of CXCL5 (magenta) in DLP sections from Pten/Trp53^(i)pe−/−^ and Pten/Trp53/Hif1a^(i)pe−/−^ mice 5 months AGI. Nuclei are stained with DAPI (white). Scale bar = 100 µm. *n* = 3 mice per group. Enlargements of boxed regions are shown on the right. **D** Proportion of CXCL5+ cells per gland in DLP sections from Pten/Trp53^(i)pe−/−^ and Pten/Trp53/Hif1a^(i)pe−/−^ mice 5 months AGI. *n* = 3 mice per group. **E** Proportion of neutrophils within CD45+ cells in DLP of Pten/Trp53^(i)pe−/−^ and Pten/Trp53/Hif1a^(i)pe−/−^ mice 5 months AGI, determined by flow cytometry as described [18]. *n* ≥ 3 animals per group. **, *p* < 0.01; Unpaired Student t-test. **F** Schematic representation of the experimental procedure investigating the effect of PX-478 treatment. IP intraperitoneal injection; OG oral gavage, Tam Tamoxifen. **G** Prostate weight (PW) of vehicle- and PX-478-treated Pten/Trp53^(i)pe−/−^ mice according to (**F**). *n* = 12 per group. * *p* < 0.05, unpaired Student t-test. **H** Proportion of immune (CD45 + ), epithelial (Epith) and stromal (Stroma) cells within lin- cells in DLP of vehicle- and PX-478-treated Pten/Trp53^(i)pe−/−^ mice, determined by flow cytometry as described [18]. *n* ≥ 8 animals per group. **, *p* < 0.01; ns, *p* ≥ 0.05, unpaired Student t-tests. **I** Proportion of neutrophils and macrophages within immune cells (CD45 + ) in DLP of vehicle- and PX-478-treated Pten/Trp53^(i)pe−/−^ mice, determined by flow cytometry, as described [18]. n ≥ 7 animals per group. *, *p* < 0.05; ns, *p* ≥ 0.05, unpaired Student t-test. **J** Representative H&E staining of DLP sections of vehicle- and PX-478-treated Pten/Trp53^(i)pe−/−^ mice. *n* ≥ 8 animals per group. Scale bar = 100 µm. Gland area (**K**) and glandular cell density (**L**) in the DLP of vehicle (V)- and PX-478-treated Pten/Trp53^(i)pe−/−^ mice. ns, *p* ≥ 0.05, Unpaired Student t-test on mean values. Small dots represent values of individual glands, large dots represent the mean per DLP.

As we previously showed that either p53 loss or HIF1 signaling enhances the progression of *Pten*-deficient prostate tumors [18, 21], we investigated whether genetic *Hif1a* inactivation impacts the progression of PTEN/p53-null PECs. Immunohistochemical analyses revealed cytoplasmic pAKT(S473) staining in most DLP epithelial cells of Pten/Trp53^(i)pe−/−^ and Pten/Trp53/Hif1a^(i)pe−/−^ mice, as well as HIF1a nuclear staining in PECs and stromal cells of Pten/Trp53^(i)pe−/−^ mice, but only in the latter in Pten/Trp53/Hif1a^(i)pe−/−^ mice (Supplementary Fig. 1B), indicating efficient inactivation of *Hif1a* in *Pten*-deficient PECs. The prostate weight in Pten/Trp53/Hif1a^(i)pe−/−^ was lower than in Pten/Trp53^(i)pe−/−^ mice 6 months AGI (Fig. 2A), but invasive and cribriform IDC were observed in both mutant mouse lines (Fig. 2B). Note that most PECs remained hypoxic after *Hif1a* inactivation, as indicated by pimonidazole staining (Supplementary Figure 1C).

We have previously shown that combined PTEN and p53 loss induces a shift from Luminal (Lum)-A to Lum-C cells, which following a crosstalk with cancer-associated fibroblasts and STAT3 signaling activation, acquire plasticity characteristics and metastatic potential (EMTc cells) [21]. Our flow cytometry and immunohistochemical analyses show that the proportion of Basal, Lum-A, Lum-C and pSTAT3+ cells was similar in tumors of Pten/Trp53^(i)pe−/−^ and Pten/Trp53/Hif1a^(i)pe−/−^ mice (Supplementary Figure 1D-F). However, the proportion of cells expressing the neutrophil chemoattractant CXCL5 [27] was reduced in the DLP of Pten/Trp53/Hif1a^(i)pe−/−^ mice (Fig. 2C, D), and accordingly, the proportion of infiltrated neutrophils was decreased (Fig. 2E). Thus, genetic inactivation of *Hif1a* in PTEN/p53-deficient PECs impairs neutrophil recruitment into hypoxic tumoral foci, but does not impact IDC formation and epithelial cell plasticity induced by *Trp53* deficiency.

### Pharmacological HIF1a inhibition remodels the immune microenvironment of primary tumors

In agreement with these results, treatment of Pten/Trp53^(i)pe−/−^ mice 5.5 months AGI with the HIF1 inhibitor PX-478 for 2 weeks (Fig. 2F) decreased prostate weight (Fig. 2G). Moreover, while it reduced immune cell infiltration (Fig. 2H), predominantly that of neutrophils (Fig. 2I), it affected neither the proportion of pSTAT3+ PECs (Supplementary Fig. 1E, F) nor tumor severity (Fig. 2J–L).

**Fig. 3:**
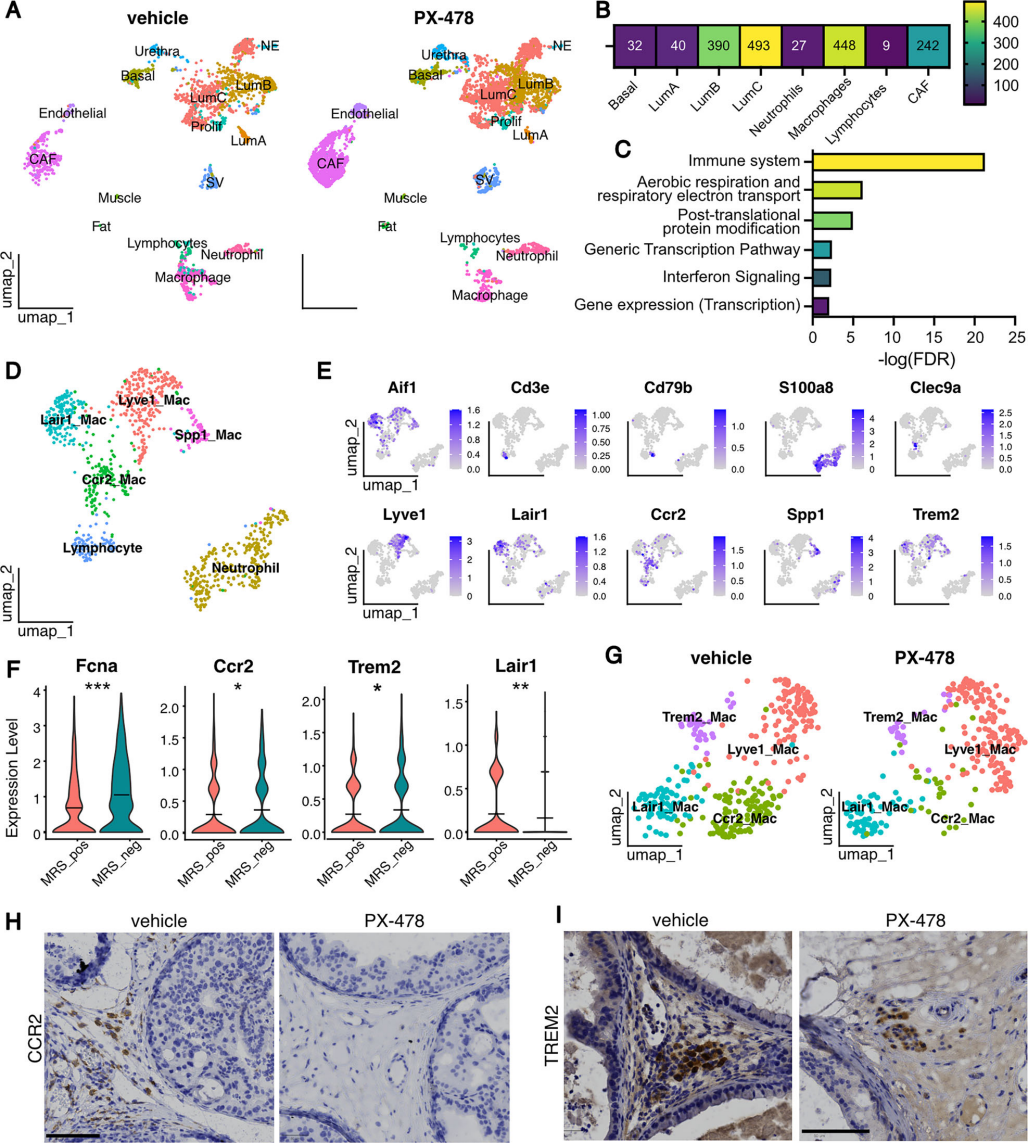
Single cell transcriptomic and histological analyses of prostates of vehicle- and PX-478-treated Pten/Trp53^(i)pe−/−^ mice. **A** Uniform Manifold Approximation and Projection (UMAP) of single cell transcriptomic analysis of dissociated DLPs of 3 vehicle- (left) and 3 PX-478-treated (right) Pten/Trp53^(i)pe−/−^ mice. Lum Luminal, CAF cancer associated fibroblasts, SV seminal vesicles, NE neuroendocrine, Prolif proliferative cells. **B** Heatmap of the number of deregulated genes in the indicated cell populations of dissociated DLP of Pten/Trp53^(i)pe−/−^ mice after PX-478 treatment. **C** Pathway over-representation analysis (ORA) of genes deregulated in LumC cells of PX-478-treated versus vehicle-treated Pten/Trp53^(i)pe−/−^ mice. FDR false discovery rate. **D** UMAP of immune cell population subclusters in DLPs of vehicle- and PX-478-treated mice. Mac macrophage. **E** Feature plot of the indicated genes in immune cell populations represented in (**D**). **F** Violin plots of the transcript levels of the indicated genes in the prostate of a treatment-naïve Pten/Trp53^(i)pe−/−^ mouse, determined by spatial transcriptomic analysis presented in Fig. 1B, and segregated by the MRS. Wilcoxon rank-sum test. * *p* < 0.05; ** *p* < 0.01; *** *p* < 0.001. **G** UMAP of immune cell population subclusters in DLPs of vehicle- and PX-478-treated Pten/Trp53^(i)pe−/−^ mice. **H** Representative immunohistochemical detection of CCR2+ cells in DLP sections of vehicle- and PX-478-treated Pten/Trp53^(i)pe−/−^ mice. *n* = 3 animals per group. Scale bar = 100 µm. **I** Representative immunohistochemical detection of TREM2+ cells in DLP sections of vehicle- and PX-478-treated Pten/Trp53^(i)pe−/−^ mice. n = 3 animals per group. Scale bar = 100 µm.

To further characterize the impact of HIF1 inhibition on the various cell types of prostatic tumors, we performed single-cell RNA seq analysis of 7267 fixed cells isolated from DLPs of Pten/Trp53^(i)pe−/−^ mice 6 months AGI, either treated with PX-478 (4984 cells) or with vehicle (2283 cells) for 2 weeks. Bioinformatic analyses identified 22 clusters, that were annotated using previously described lineage markers [18, 21, 28] (Supplementary Fig. 2A–C). Epithelial cells represented 58.4% of the fixed cells, stromal cells 18% and immune cells 12.2%. Among epithelial populations, we identified Basal (*Krt5*) cells (cluster 8) and 3 Luminal subpopulations [LumA (*Tgm4, Glb1l3;* cluster 18), LumB (*Spink1, Trpv6*; clusters 4-5) and LumC (*Krt4, Tacstd2*; clusters 0, 1, 6, 10) cells] (Supplementary Fig. 2B, C). Moreover, epithelial cells sub-clustering identified cells with an enriched EMTc signature [21] (Supplementary Fig. 2D, E). Rare neuroendocrine cells [NE] (*Syp*) were also identified (Supplementary Fig. 2A–C; cluster 21). Cluster 15 expresses markers of luminal (*Krt8)* and basal (*Krt5)* cells, as well as *Aqp3*, and according to Crowley et al. [29], was annotated as Urethral cells. Cluster 19 expresses *Adipoq*, a marker of adipocytes, and cluster 20 expresses *Tpm2*, and thus most probably corresponds to muscle cells (Supplementary Fig. 2A–C) [30]. All the above-described cell populations were identified in prostates of vehicle- and PX-478-treated Pten/Trp53^(i)pe−/−^ mice (Fig. 3A). In both conditions, 25% of LumC cells express the EMTc signature (Supplementary Fig. 2F), in line with a similar abundance of pSTAT3+ cells (Supplementary Fig. 1E, F). Note that the proportion of epithelial, stromal and immune cells in our previous single-cell RNA seq analysis of non-fixed cells isolated from a prostate of Pten/Trp53^(i)pe−/−^ mice 6 months AGI was 10%, 28.8% and 55%, respectively [21], showing that fixation results in an enrichment of epithelial cells.

Differential gene expression analyses revealed that luminal epithelial cells and macrophages are the most affected by PX-478 treatment (Fig. 3B, Supplementary Table 4). In addition, over-representation analysis of the deregulated genes in Lum-C cells showed an enrichment of those associated with generic transcription pathways, as well as with the immune system and inflammation (Fig. 3C). Moreover, upon PX-478 treatment, macrophages displayed an activation of antigen presentation and complement cascade, as well as a decrease in interleukin-1 signaling (Supplementary Fig. 2G; Supplementary Tables 5–6), indicating an anti-tumoral polarization.

Reclustering and annotation of immune cell populations identified a cluster of lymphocytes with T- (*Cd3e*) and B-cells (*Cd79b*), a neutrophil cluster (*S100a8, S100a9)*, few dendritic cells (*Clec9a*), and 4 clusters of macrophages (*Aif1*) (Fig. 3D and Supplementary Fig. 2H). The macrophage clusters include: (i) *Lyve1/Folr2/Timd4/Fcna*+ perivascular macrophages [Lyve1+ macrophages] [31]; (ii) *Ccr2+* macrophages/monocytes [31]; (iii) *Trem2/Spp1*+ macrophages [32, 33] [Trem2+ macrophages]; and (iv) Lair1*+* macrophages, coexpressing *Irf8* and *Ccl4* [34] (Fig. 3D, E, Supplementary Fig. 2H). Interestingly, spatial transcriptomic analysis showed that *Lair1* expression was higher in regions with a positive MRS score (Fig. 3F), indicating that these macrophages localize in tumor areas. In contrast, *Trem2*, *Fcna,* and *Ccr2* expression was higher in regions with a negative MRS score (Fig. 3F), indicating that Trem2 + , Lyve1+ and Ccr2+ cells are located in the stroma, in line with our previous study [33].

Strikingly, the proportion of Ccr2+ cells was markedly decreased upon PX-478 treatment, while that of the other macrophage subclusters was almost not affected (Fig. 3G). Immunohistochemical analyses revealed numerous CCR2+ cells in the stroma of vehicle-treated Pten/Trp53^(i)pe−/−^ mice, and very few in prostates of PX-478-treated ones (Fig. 3H). In contrast, TREM2+ cells, which have been shown to promote PCa progression [12, 13, 32, 33], were detected in the stroma of vehicle- and PX-478-treated animals (Fig. 3I). Together, these results show that systemic HIF1 inhibition decreases the number of stromal CCR2+ myeloid cells in primary tumors, but has little effect, if any, on TREM2+ macrophages.

### Systemic HIF1 inhibition eliminates liver metastatic niches

**Fig. 4:**
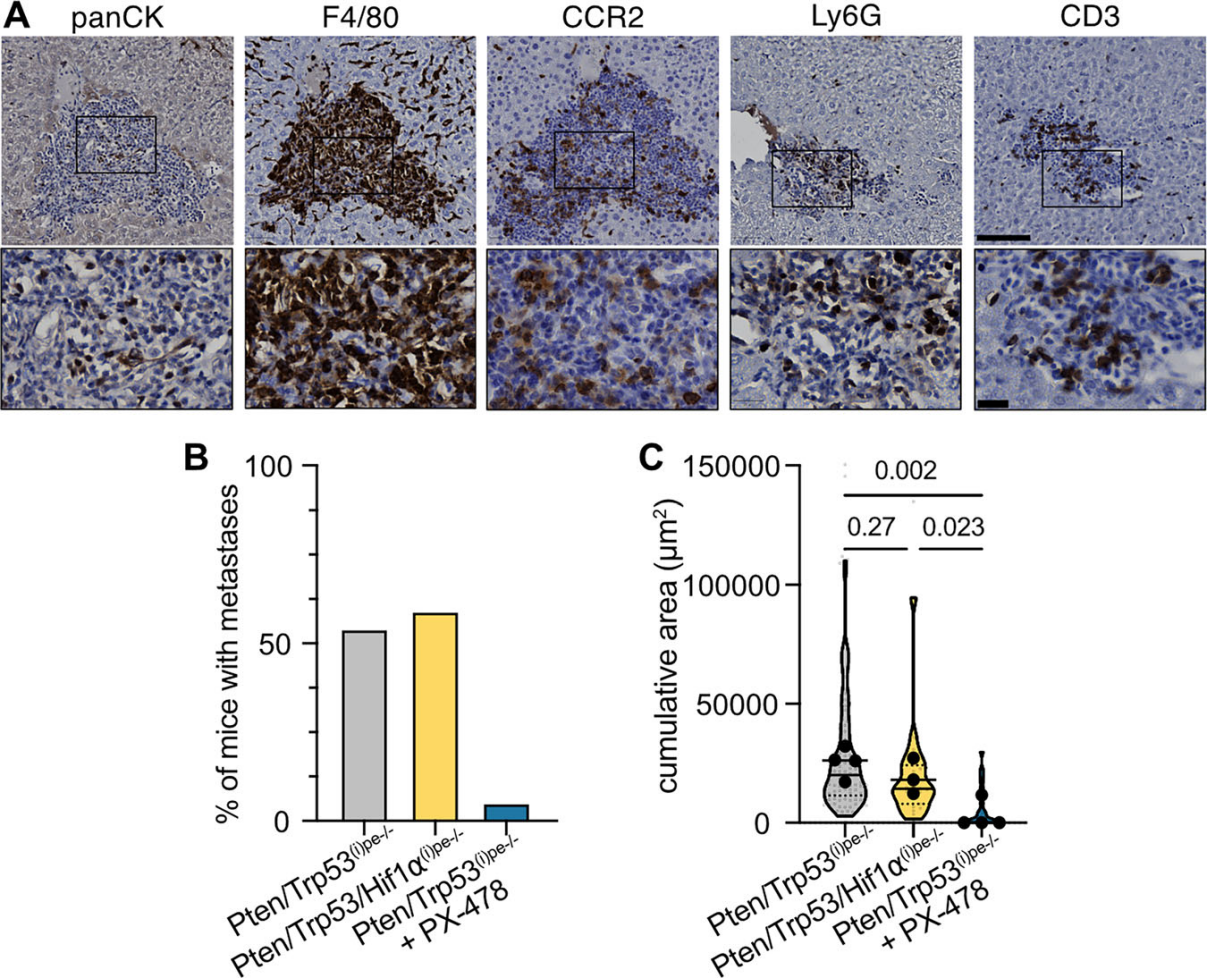
Analysis of hepatic micro-metastases. **A** Representative immunohistochemical detection of the indicated proteins in livers of Pten/Trp53^(i)pe−/−^ mice. n ≥ 4 animals. Scale bar = 100 µm. Enlargements of boxed regions are shown below. **B** Proportion of vehicle- and PX-478- treated Pten/Trp53^(i)pe−/−^ mice, and of Pten/Trp53/Hif1a^(i)pe−/−^ mice, with liver micro-metastases. **C** Cumulative area of metastatic infiltrates per liver section of vehicle- and PX-478- treated Pten/Trp53^(i)pe−/−^ mice, and of Pten/Trp53/Hif1a^(i)pe−/−^ mice. Violin plots represent the distribution of values of individual infiltrates; large dots represent the mean per animal. *n* ≥ 3 animals per group. Unpaired Student t-test.

We have previously shown that prostatic tumors of Pten/Trp53^(i)pe−/−^ mice spread to the liver at a 50% penetrance [21]. Immunostaining of hepatic infiltrates of Pten/Trp53^(i)pe−/−^ mice unraveled a high number of macrophages (F4/80 + ), and the presence of CCR2+ myeloid cells, neutrophils (Ly6G + ) and T-cells (CD3 + ) (Fig. 4A). As PX-478 treatment reduced neutrophil and CCR2+ myeloid cell infiltration in the primary tumor, we explored the impact of impaired systemic HIF1 signaling on hepatic infiltrates. Micro-metastases were detected only in 1/20 Pten/Trp53^(i)pe−/−^ mice after 2 weeks of PX-478, but in 11/21 Pten/Trp53/Hif1a^(i)pe−/−^ mice (Fig. 4B). Note that the area of the infiltrated liver was similar in Pten/Trp53^(i)pe−/−^ and Pten/Trp53/Hif1a^(i)pe−/−^ mice (Fig. 4C). Thus, PTEN- and p53-null PECs do not rely on cell-intrinsic HIF1 signaling for metastatic dissemination, whereas micro-metastases are highly sensitive to systemic Hif1a inhibition.

**Fig. 5:**
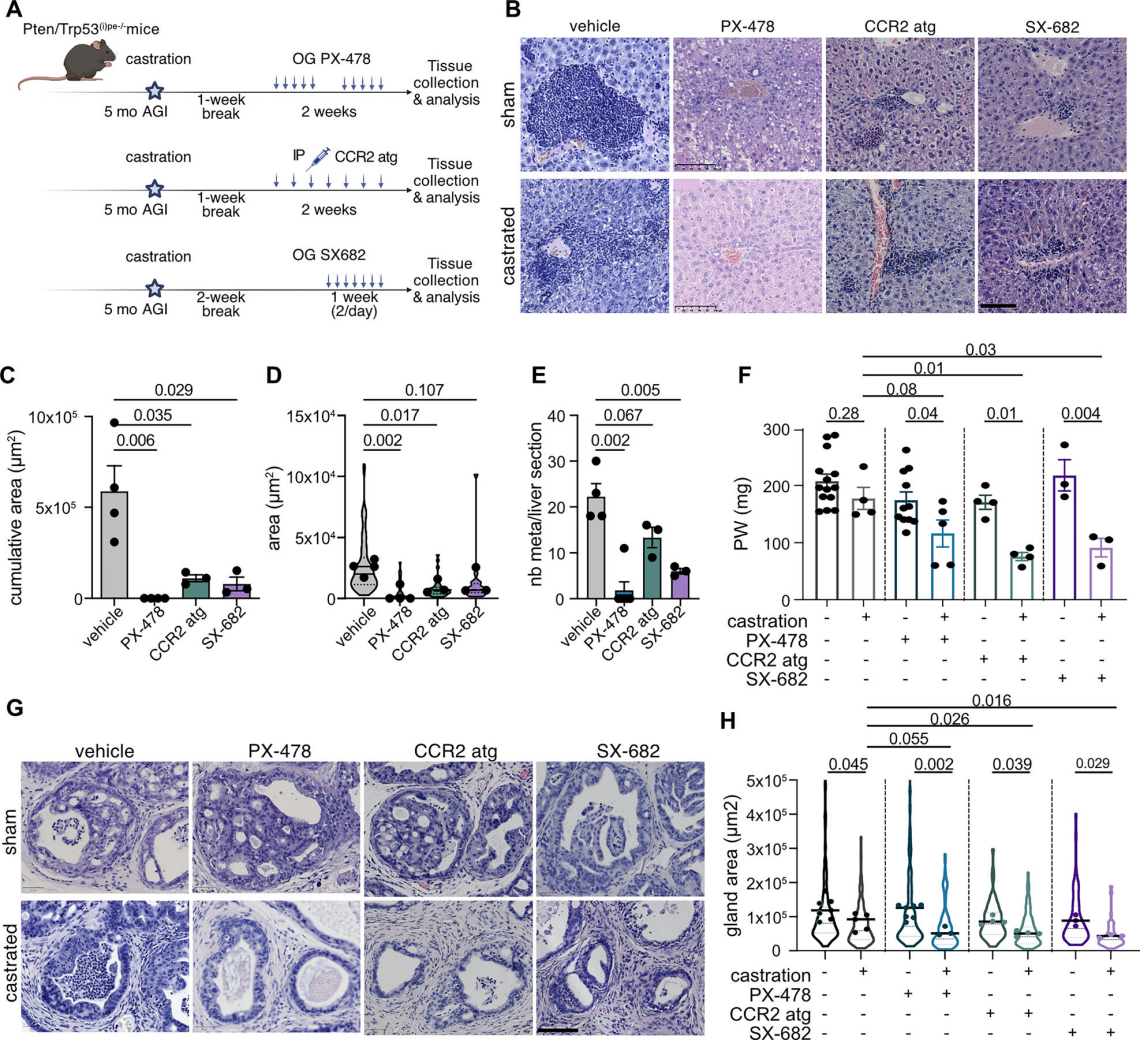
Effect of various treatments on metastasis and castration resistance of Pten/Trp53^(i)pe−/−^ mice. **A** Schematic representation of the experimental procedure investigating the effect of PX-478, CCR2 antagonist or SX-682 treatment in Pten/Trp53^(i)pe−/−^ mice. IP intraperitoneal injection, OG oral gavage. **B** Representative Hematoxylin-Eosin staining of liver sections of sham-operated (sham) and castrated Pten/Trp53^(i)pe−/−^ mice treated with vehicle, PX-478, CCR2 antagonist, or SX-682. *n* ≥ 2 mice per group. Scale bar = 100 µm. **C** Cumulative area of metastatic infiltrates per liver section of Pten/Trp53^(i)pe−/−^ mice treated with vehicle, PX-478, CCR2 antagonist, or SX-682. *n* ≥ 3 animals per group. Unpaired Student t-test on the mean values. **D** Area of metastatic infiltrates in a liver section of Pten/Trp53^(i)pe−/−^ mice treated with vehicle, PX-478, CCR2 antagonist, or SX-682. Violin plots represent the distribution of values of the infiltrates, and large dots represent the mean area. *n* ≥ 3 animals per group. Unpaired Student t-test. **E** Number of metastatic infiltrates per liver section of Pten/Trp53^(i)pe−/−^ mice treated with vehicle, PX-478, CCR2 antagonist or SX-682. *n* ≥ 3 animals per group. Unpaired Student t-test. **F** Prostate weight (PW) of vehicle-, PX-478-, CCR2 antagonist- or SX-682-treated, sham-operated (-) and castrated (+) Pten/Trp53^(i)pe−/−^ mice. *n* ≥ 5 per group. Two-way ANOVA with post-hoc Tukey. **G** Representative Hematoxylin-Eosin staining of prostate sections of vehicle-, PX-478-, CCR2 antagonist- or SX-682-treated, sham-operated (sham) and castrated Pten/Trp53^(i)pe−/−^ mice. *n* ≥ 3 per group. Scale bar = 100 µm. **H** Gland area in the DLP of vehicle-, PX-478-, CCR2 antagonist- or SX-682-treated, sham-operated (-) and castrated (+) Pten/Trp53^(i)pe−/−^ mice. *n* ≥ 3 per group. Two-way ANOVA with post-hoc Tukey on mean values. Violin plots represent the distribution of glands’ individual values; large dots represent mean per DLP.

In addition, treatment with either the CXCR2 antagonist SX-682 [35] or CCR2 antagonist [36], impeding neutrophil and monocyte chemotaxis, respectively, strongly reduced the metastatic load in the liver (Fig. 5A–C). Interestingly, while CCR2 antagonist reduced the size of metastatic niches, SX-682 mainly reduced their number (Fig. 5D, E). These results show that neutrophils and CCR2+ cells contribute to the maintenance of metastatic niches. Note that combined inhibition of these populations was not more potent than individual ones (Supplementary Fig. 3A–C), whereas systemic inhibition of HIF1 targets metastatic disease with an increased efficiency (Fig. 5B–E).

### Pharmacological HIF1 inhibition sensitizes PTEN/p53-deficient prostatic tumors to androgen deprivation

Castration of Pten/Trp53^(i)pe−/−^ mice 5 months AGI had almost no impact on prostate weight and tumor histology 1 month after surgery (Fig. 5F–H). Moreover, metastatic liver infiltrates were observed in 3 out of 7 castrated Pten/Trp53^(i)pe−/−^ mice (Fig. 5B), indicating that disseminated PECs are also castration-resistant.

As targeting HIF1 signaling in Pten-deficient prostate tumors alleviates castration resistance of primary tumors [19], we wondered whether it would have similar effects on PTEN/p53-deficient tumors. A two-week PX-478 treatment of castrated mice decreased prostate weight and induced a significant primary tumor shrinkage (Fig. 5F–H). Thus, pharmacological HIF1 signaling inhibition overcomes androgen deprivation resistance of PTEN/p53-deficient primary prostate tumors.

As previous studies reported that neutrophils and CCR2+ myeloid cells contribute to androgen-deprivation resistance [10, 37], we treated castrated Pten/Trp53^(i)pe−/−^ mice either with SX-682 or the CCR2 antagonist used above. Both treatments did not impact the prostate weight of sham operated mice, but decreased that of castrated ones (Fig. 5F). Moreover, histological analyses revealed a shrinkage of prostatic glands in castrated animals treated with either SX-682 or the CCR2 antagonist (Fig. 5G, H). Thus, neutrophils and CCR2+ myeloid cells contribute to castration resistance of primary prostate tumors.

Importantly, treatment with PX-478, or with either SX-682 or the CCR2 antagonist, eliminated or strongly reduced the size of liver micro-metastases, respectively, as in non-castrated mice (Fig. 5B). Thus, metastatic niches are sensitive to those treatments even in androgen-deprived conditions.

## Discussion

Efficient therapies have been developed for localized PCa, but metastatic forms are resistant to current treatments, and remain a major clinical issue. Thus, the elucidation of the molecular and cellular events contributing to the metastatic dissemination and resistance to current treatments of aggressive prostate tumors is of major importance to identify new therapeutic targets.

We previously generated Pten/Trp53^(i)pe−/−^ mice, in which both *Pten* and *Trp53* are selectively inactivated in prostatic luminal cells at adulthood [20]. Those PECs give rise to PINs, undergo plasticity, form IDC, and disseminate to the liver [21]. In this study, using Pten/Trp53^(i)pe−/−^ mice as a model of de novo metastatic PCa, we further investigated the signaling pathways and cell types promoting tumor progression to identify new therapeutic vulnerabilities for aggressive PCa.

In PCa patients, tumor hypoxia correlates with high Gleason scores, tumor invasiveness [38] and accelerated relapse [39, 40]. Our results show that early PIN lesions and advanced tumors of Pten/Trp53^(i)pe−/−^ mice are hypoxic, and that HIF1 signaling is enhanced in PECs, as previously observed in Pten^(i)pe−/−^ mice [18]. Our spatial transcriptomic analyses revealed that hypoxic tumors are infiltrated by neutrophils, in line with previously reported strong infiltration of myeloid immunosuppressive cells in human prostatic tumors [41, 42].

To characterize the role of HIF1 signaling in PECs in the progression of PTEN/p53-deficient tumors, we analyzed Pten/Trp53/Hif1a^(i)pe−/−^ mice in which *Hif1a* is inactivated in PECs in addition to *Pten* and *Trp53*. Impaired HIF1 signaling in PECs did not alter PIN formation, the proportion of pSTAT3+ cells and hepatic infiltrates. However, *Hif1a*-deficient PECs expressed less of the neutrophil chemoattractant CXCL5, and the proportion of neutrophils was decreased among infiltrated immune cells. Thus, epithelial HIF1 plays a key role in local neutrophil chemotaxis, but is not required for p53 loss-induced PECs plasticity and metastatic spreading.

Pharmacological inhibition of HIF1 with PX-478 in Pten/Trp53^(i)pe−/−^ mice decreased neutrophil and CCR2+ myeloid cell infiltration in primary tumors, but did not affect PEC plasticity and tumor severity. Importantly, it eradicated the liver micro-metastases, in contrast to HIF1a inactivation in PECs of Pten/Trp53^(i)pe−/−^ mice, indicating that HIF1 signaling in non-epithelial cells plays a central role in the maintenance of the metastatic niche. Impaired neutrophil and/or monocyte chemotaxis with the CXCR2 antagonist SX-682 [35] or the CCR2 antagonist [36], respectively, also reduced the metastatic load in the liver, but with lower efficiencies. Thus, additional cell types or signaling pathways targeted by PX-478 most probably contribute to sustain the metastatic niche. It is unlikely that this impact on metastases results from PX-478- induced off-target effects, as none were reported since its initial characterization [43, 44]. As HIF1a is a known regulator of neutrophil viability [45], PX-478 might directly impact these cells, but whether it similarly impairs CCR2+ myeloid cells remains to be determined. Note that *Ccr2* promoter encompasses an HIF1 response element [46], suggesting that the expression of this receptor is reduced upon PX-478 treatment.

Several groups recently reported that Trem2 + /Spp1+ macrophages are associated with reduced progression-free survival and bone metastasis in patients [32], as well as poor response to immunotherapies [47]. Our results show that PX-478 treatment does not affect the number of infiltrated Trem2+ macrophages in the stroma of *Pten/Trp53*-deficient tumors, indicating that this population is not dependent on HIF1 signaling for their maintenance in tumors. Whether hypoxic niches contribute to their recruitment remains unknown.

Previous studies highlighted the contribution of myeloid cells, including neutrophils [10, 11] and macrophages [13, 48, 49] in the development of castration-resistant PCa. Moreover, several studies performed in xenograft models with an incomplete immune system reported the contribution of CCR2+ cells to androgen-deprivation resistance and metastasis [37, 48]. We demonstrate that inhibition of either neutrophils or CCR2+ myeloid cells induces primary tumor regression in castrated Pten/Trp53^(i)pe−/−^ mice, and impacts metastatic niches. Moreover, combined inhibition of those populations also reduced, but did not eradicate liver infiltrates. In contrast, pharmacological inhibition of HIF1a signaling overcomes castration resistance and eradicates liver metastasis.

## Conclusion

Collectively, our data shed light on how the presence of hypoxic niches shapes therapy resistance of primary prostatic tumors through the recruitment of tumor-sustaining myeloid populations. Importantly, we provide evidence that pharmacological inhibition of HIF1 signaling represents a more potent therapeutic strategy against metastatic and hormone-refractory prostate cancer than those targeting myeloid chemotaxis.

## Supplementary information

Supplementary material

Supplementary Tables

Supp F1

Supp F2

Supp F3

Related Manuscript File

## Data Availability

All data needed to evaluate the conclusions in the paper are present in the paper and/or the Supplementary Materials. Sequencing data are available in the GEO repository under the accession number GSE303530. The mouse lines are available on request.
