# Supplementary material for: HIF1 inhibition targets tumoral and myeloid cells, and is a promising therapy for metastatic castration-resistant prostate cancer
Source: Cell Death Dis. 2026 Mar 27;17(1):420. doi: 10.1038/s41419-026-08590-8 (PMC13149874; doi:10.1038/s41419-026-08590-8)
Supplement: Supplementary file 1 — Supplementary material [file 41419_2026_8590_MOESM1_ESM.docx]

**HIF1 inhibition targets tumoral and myeloid cells, and is a promising therapy for metastatic castration-resistant prostate cancer**

Darya Yanushko^1, 2, 3, 4^, Angélique Pichot^1, 2, 3, 4^, Alexandre Vincent^1, 2, 3, 4^, Céline Keime^1, 2, 3, 4^, Gilles Laverny^1, 2, 3, 4*^ and Daniel Metzger^1, 2, 3, 4*^

**Supplementary material**

**Supplementary Fig.1. Impact of genetic and pharmacological inhibition of Hif1a on tumors of Pten/Trp53^(i)pe-/-^ mice**

1. H&E staining of the prostate section of a Pten/Trp53^(i)pe-/-^ mouse used for spatial transcriptomics. The anterior (AP, red), dorsolateral (DLP, yellow) and ventral (VP, green) prostate are marked. Data from urethra and ducts were excluded from the analysis.
2. Representative immunohistochemical detection of pAKT (S473) and HIF1a in Pten/Trp53^(i)pe-/-^ and Pten/Trp53/Hif1a^(i)pe-/-^ mice 3 months AGI. N=3 animals per group. Inset, higher magnification of the boxed area. Scale bar = 100 µm.
3. Representative immunohistochemical detection of pimonidazole in Pten/Trp53/Hif1a^(i)pe-/-^ mice 6 months AGI. N≥3 animals per group. Scale bar = 100 µm.
4. Proportion of Luminal-A (LumA), Luminal-C (LumC) and Basal cells within PECs in Pten/Trp53^(i)pe-/-^ and Pten/Trp53/Hif1a^(i)pe-/-^ mice 5 months AGI determined by flow cytometry, as described ^18^. N≥4 animals per group. ns, p≥0.05, unpaired Student t-tests.
5. Representative immunohistochemical detection of pSTAT3 in DLPs of vehicle- and PX-478-treated Pten/Trp53^(i)pe-/-^ mice and of Pten/Trp53/Hif1a^(i)pe-/-^ mice 5 months AGI. N≥3 animals per group. Inset, higher magnification of the boxed area. Black arrows point to pSTAT3+ cells. Scale bar = 100 µm.
6. Quantification of pSTAT3+ cells in DLP glands of vehicle- and PX-478-treated Pten/Trp53^(i)pe-/-^ mice, and of Pten/Trp53/Hif1a^(i)pe-/-^ mice 5 months AGI. Small dots represent values in individual glands, large dots represent mean values per DLP. ns, p≥0.05, one-way ANOVA on mean values.

**Supplementary Fig. 2. Single-cell analyses of DLPs of vehicle- and PX-478 -treated Pten/Trp53^(i)pe-/-^ mice**

1. Uniform Manifold Approximation and Projection (UMAP) of single cell transcriptomic analysis of dissociated DLPs of vehicle- and PX-478-treated Pten/Trp53^(i)pe-/-^ mice.
2. DotPlot of lineage marker expression in the cell populations depicted in A.
3. UMAP of annotated cell populations. Lum, Luminal; CAF, cancer associated fibroblasts ; SV, seminal vesicles ; NE, neuroendocrine ; Prolif ; proliferative cells.
4. UMAP of epithelial cell populations subclustering.
5. EMTc signature-expressing cells (red) in epithelial cells depicted in D.
6. EMTc signature-expressing cells (red) in epithelial cells of the DLP of vehicle-treated and PX-478-treated Pten/Trp53^(i)pe-/-^ mice.
7. Pathway over-representation analysis (ORA) of genes downregulated (blue) and upregulated (yellow) in macrophages upon PX-478 treatment. FDR – false discovery rate.
8. DotPlot of gene expression in immune cell populations.

**Supplementary Fig.3. Effect of combinatorial SX-682 and CCR2 antagonist treatment on liver metastases in Pten/Trp53^(i)pe-/-^ mice.**

1. Number of metastatic infiltrates per liver section of Pten/Trp53^(i)pe-/-^ mice treated with CCR2 antagonist, SX-682, or the combination of the latter. Dashed line indicates the mean value of vehicle-treated animals. n≥ 3 animals per group. Unpaired Student t-test.
2. Area of metastatic infiltrates in liver sections of Pten/Trp53^(i)pe-/-^ mice treated with CCR2 antagonist, SX-682, or the combination of the latter. Violin plots represent the distribution of values of individual infiltrates, large dots represent the mean per animal. n≥ 3 animals per group. Unpaired Student t-test.
3. Cumulative area of metastatic infiltrates per liver section of Pten/Trp53^(i)pe-/-^ mice treated with vehicle, PX-478, CCR2 antagonist, SX-682, or the combination of the latter. Dashed line indicates the mean value of vehicle-treated animals. n≥ 3 animals per group. Unpaired Student t-test.

**Supplementary Tables.**

**Supplementary Table 1.** Differentially expressed genes (DEGs) between MRS-low and MRS-high regions.

**Supplementary Table 2.** Pathway over-representation analysis (ORA) of genes enriched in MRS-high regions.

**Supplementary Table 3.** ORA of genes enriched in MRS-low regions.

**Supplementary Table 4.** DEGs in cell populations of prostates of Pten/Trp53^(i)pe-/-^ mice treated with vehicle or with PX-478.

**Supplementary Table 5.** ORA of genes upregulated in macrophages upon PX-478 treatment.

**Supplementary Table 6.** ORA of genes downregulated in macrophages upon PX-478 treatment.
