# Supplementary figures and images for: HIF1 inhibition targets tumoral and myeloid cells, and is a promising therapy for metastatic castration-resistant prostate cancer
Source: Cell Death Dis. 2026 Mar 27;17(1):420. doi: 10.1038/s41419-026-08590-8 (PMC13149874; doi:10.1038/s41419-026-08590-8)

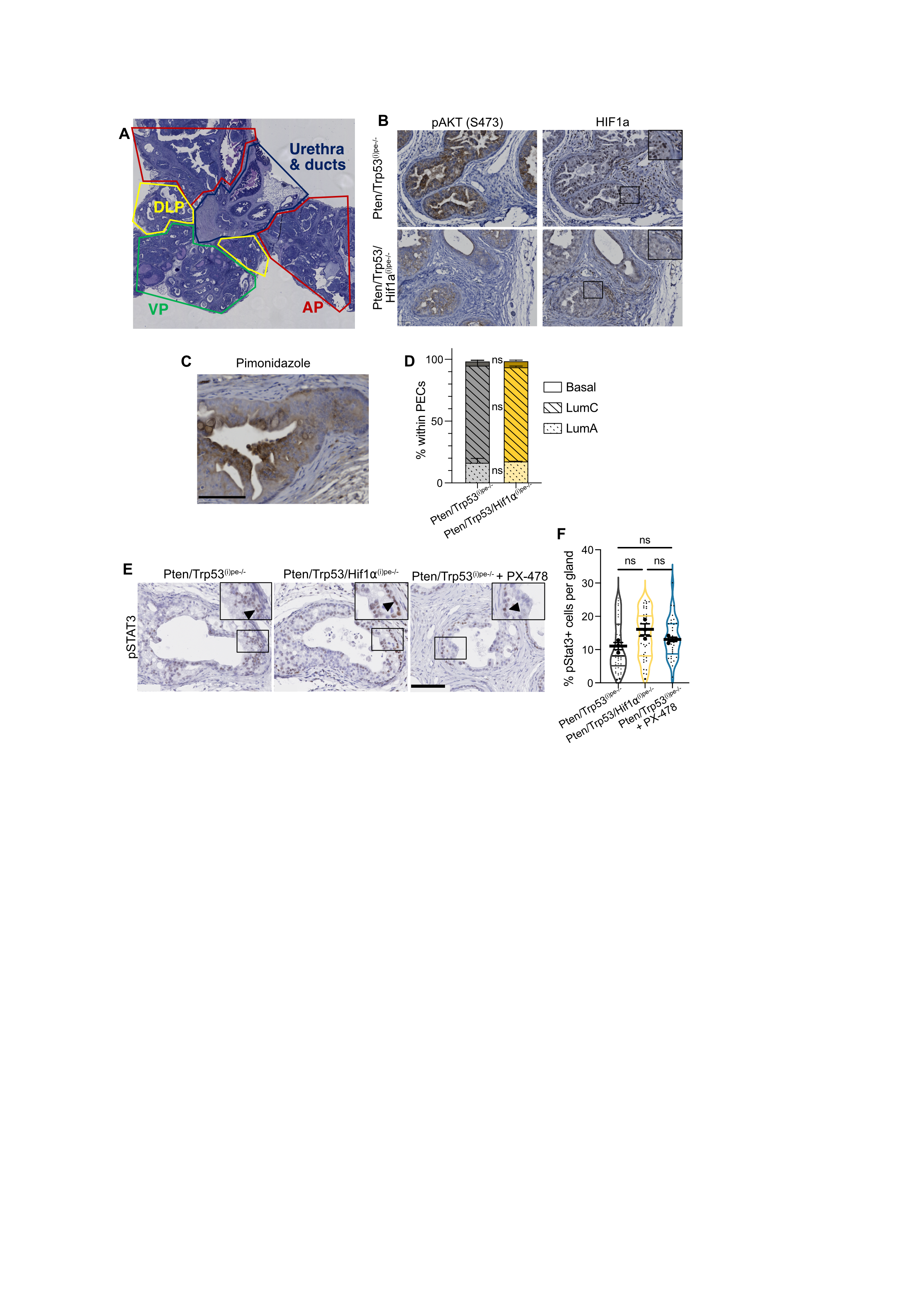

Supplement: Supplementary file 3 — Supp F1 [file 41419_2026_8590_MOESM3_ESM.tif]

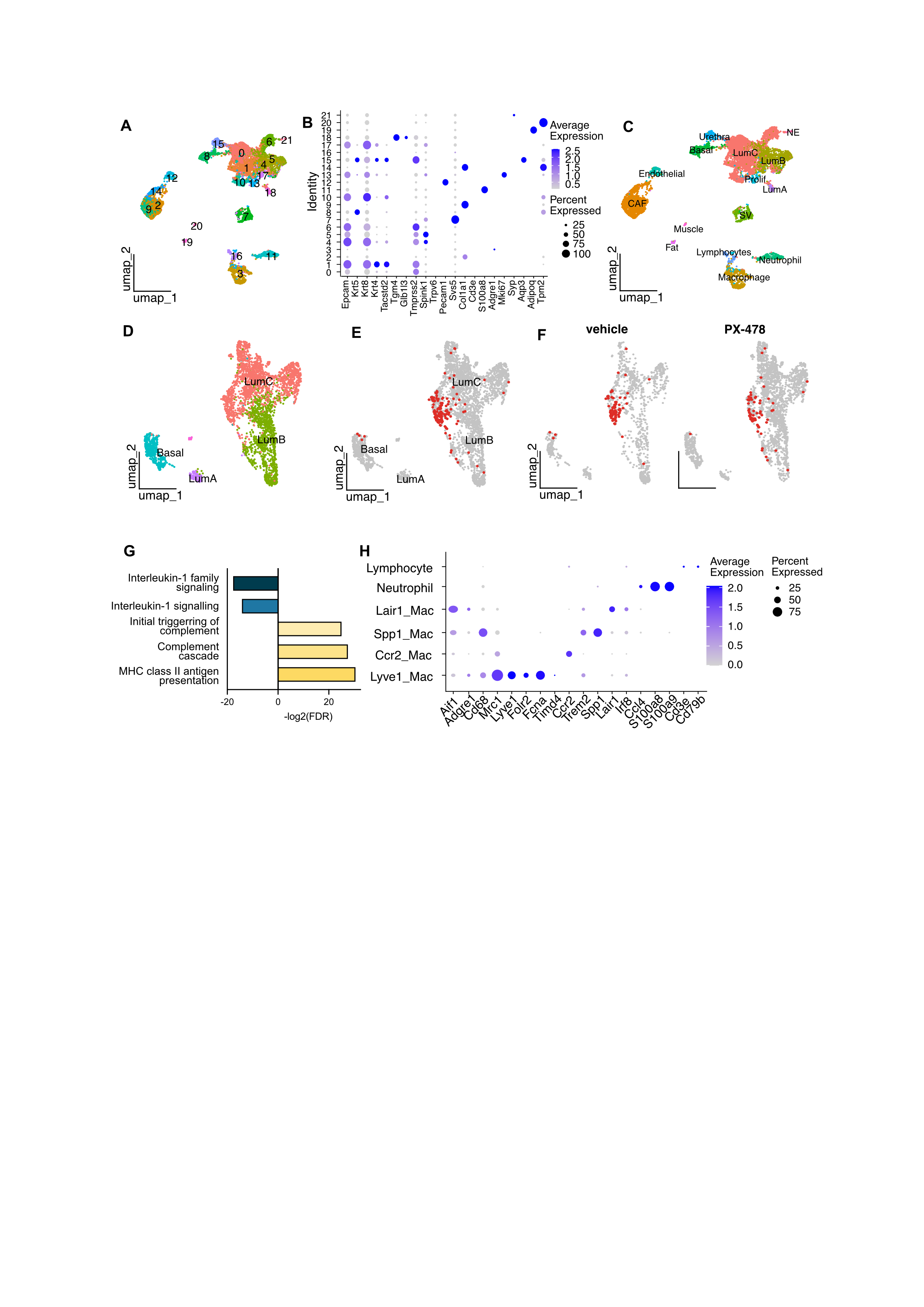

Supplement: Supplementary file 4 — Supp F2 [file 41419_2026_8590_MOESM4_ESM.tif]

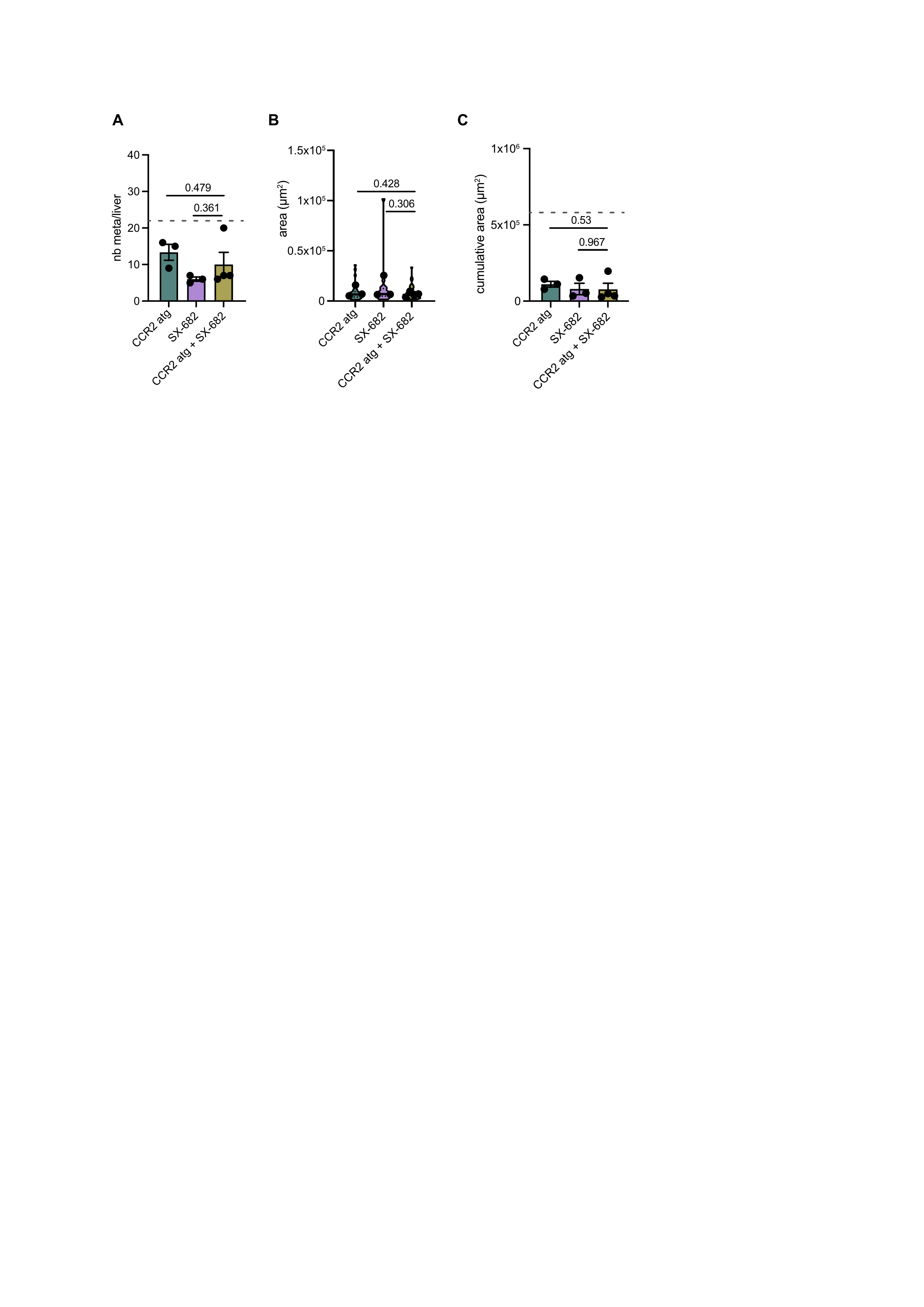

Supplement: Supplementary file 5 — Supp F3 [file 41419_2026_8590_MOESM5_ESM.tif]
